# Infectious Endocarditis Presenting as Intracranial Hemorrhage in a Patient Admitted for Lumbar Radiculopathy

**DOI:** 10.1155/2011/428729

**Published:** 2011-07-24

**Authors:** David Ethan Kahn, Kristine O'Phelan, Ross Bullock

**Affiliations:** ^1^Miller School of Medicine University of Miami, Department of Neurology, Clinical Research Building, 1120 NW 14th Street, 13th Floor, Miami, FL 33136, USA; ^2^Jackson Memorial Hospital, Department of Neurosurgery, 1095 NW 14th Ter No. 2, Miami, FL 33136-1060, USA

## Abstract

Infectious endocarditis is frequently found in the neurologic intensive care unit and may rarely be the cause of intracranial hemorrhage. In such instances, further diagnostic imaging to search for an underlying structural lesion is prudent. Well-known causes of these hemorrhages include cardioembolism with hemorrhagic transformation, septic emboli, and mycotic aneurysms. We present a case of a patient who was admitted for routine evaluation and pain management of lumbar radiculopathy, who developed a large intraparenchymal hemorrhage and was found to have bacterial endocarditis. This was diagnosed retrospectively from positive hematoma cultures and a vegetation on transesophageal echocardiogram. Further evaluation revealed a mycotic aneurysm.

## 1. Introduction

Infectious endocarditis commonly presents with a multitude of neurologic sequelae. These include encephalopathy, headache, seizures, stroke, and meningitis. Stroke in the setting of infectious endocarditis (IE) may be hemorrhagic or ischemic and is commonly secondary to cardioembolism, septic emboli, or mycotic aneurysms. The incidence of intracranial hemorrhage is uncommon, occurring in 2.7–7% of patients with IE [1]. This may present as subdural hematoma, subarachnoid hemorrhage, or intraparenchymal hemorrhage. Intracranial hemorrhage is most commonly caused by mycotic aneurysms, which account for 5% of neurologic complications associated with IE. [2] Rupture of mycotic aneurysms is associated with 80% mortality [1]. We present a case of 72-year-old male admitted for severe back pain who developed a massive intracranial hemorrhage and was found to have a mycotic aneurysm.

## 2. Case Report

**Figure 1:**
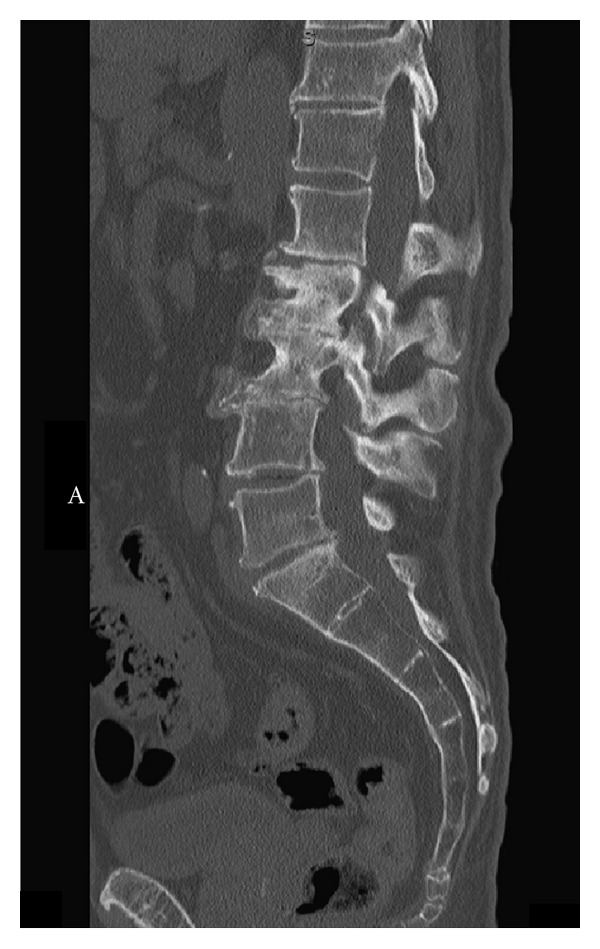
Sagittal view of the lumbar spine on CT reveals a calcified L2-L3 disc bulge with narrowing of the intervertebral disc space.

**Figure 2:**
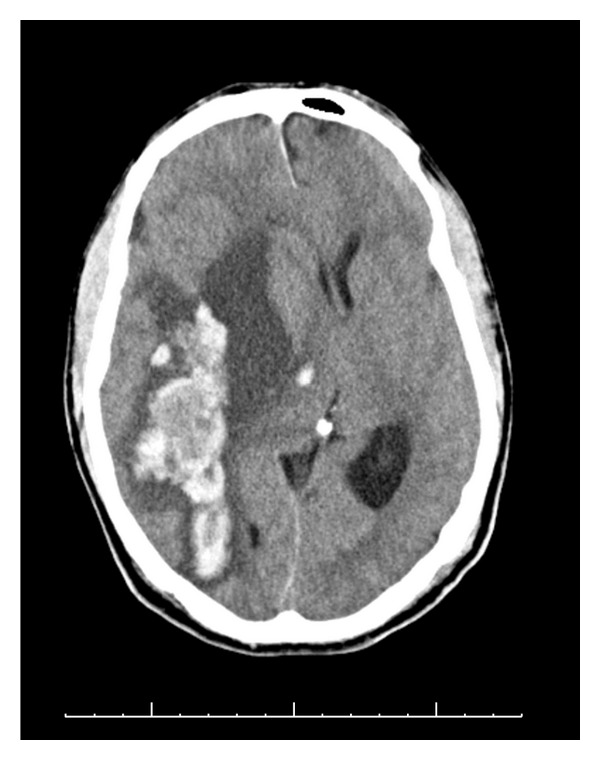
CT of the head, performed within 30 minutes of presentation, reveals a large intraparenchymal hemorrhage in the right cerebral hemisphere involving the right parietotemporal lobes. There is transfalcine herniation and a right to left midline shift measuring 1.9 cm.

**Figure 3:**
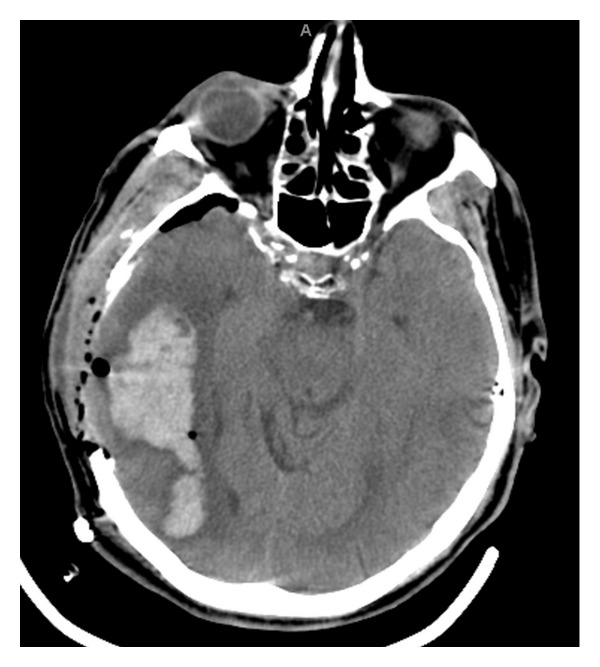
CT of the head immediately after a right sided craniotomy was performed.

A 72-year-old male was admitted for diagnosis and management of severe sudden onset lumbar back pain that radiated down both legs to the top of his feet. Subtle weakness in the L4/5 distribution was noted on exam, though muscle bulk was preserved. Prior to admission, he had been treated for possible discitis at an outside hospital with 2 weeks of empiric antibiotic coverage. A CT scan of the lumbar spine, performed on admission, revealed multilevel disc changes at the levels of L1-2, L2-3, and L5-S1 (Figure 1). Bilateral renal hypodensities were incidentally seen, although renal function was well preserved. Two days after admission, immediately after a routine preoperative chest X-ray, the patient was noted to become acutely less responsive, and began posturing in the right upper extremity with ipsilateral head turning, followed by plegia of the left arm. Initially, he was able to follow simple commands, but within 30 seconds, he stopped doing so, and bilateral decerebrate posturing was noted. His pupils at this time were 4 mm and unreactive on right and 2 mm and unreactive on left. His blood pressure was 148/62. Mannitol was administered, the patient was intubated, and a stat CT of the head was done. It revealed a right parieto-temporal and right thalamic hemorrhage, with a 1.9 cm midline shift. Subfalcine and right uncal herniation were present (Figure 2). The patient was taken to the operating room for an emergent frontotemporal craniectomy with duraplasty (Figure 3). During the procedure, a hematoma containing multiple abnormal rubbery pinkish colored nodules was noted. A specimen was sent for pathologic examination, and culture of the hematoma was also sent. Subsequently, the patient was transferred to the neurosurgical intensive care unit. He had now developed midposition pupils and no response to noxious stimulation; however, his gag and corneal reflexes were preserved.

 At this time, diagnostic considerations included a stroke with hemorrhagic conversion, amyloid angiopathy, or an underlying tumor (i.e., hemorrhagic metastasis associated with possible renal cell carcinoma). Pathology of the rubbery nodules was found to have blood clots with fragments of brain tissue infiltrated with polymorphonuclear lymphocytes, consistent with acute brain inflammation. 

 The patient was empirically started on vancomycin and cefepime for discitis. Elevated ICP and herniation syndrome were treated with Mannitol and 48 hours of therapeutic hypothermia achieved with surface cooling via the Arctic sun (Medivance) device set to a target temperature of 34°C (while therapeutic hypothermia may be used for refractory intracranial hypertension, it is not considered standard of care). He regained his pupillary response, and his motor exam improved slightly to decerebration on the left.

 An MRI of the brain did not show an underlying lesion within the hematoma. Blood cultures attained 2 days after injury for a low grade fever were negative, and antibiotics were eventually stopped due to low suspicion for discitis. One week after onset, he developed a temperature of 38.6°C. Broad-spectrum antibiotics were restarted. The following day cultures taken from the hematoma exhibited light growth of *Streptococcus viridans*. Repeat blood cultures were immediately drawn and revealed no growth. A normal ejection fraction without evidence of vegetation was present on transthoracic echocardiogram. Subsequent transesophageal echocardiogram exhibited a 9 mm mobile echodensity on the aortic valve leaflet. C-reactive protein was 19.4 mg/dL, leukocyte count 16,000 L, and ESR 83 mm/hr. The patient was diagnosed with subacute bacterial endocarditis and started on penicillin 4 million units q4 hours for a six-week course.

**Figure 4:**
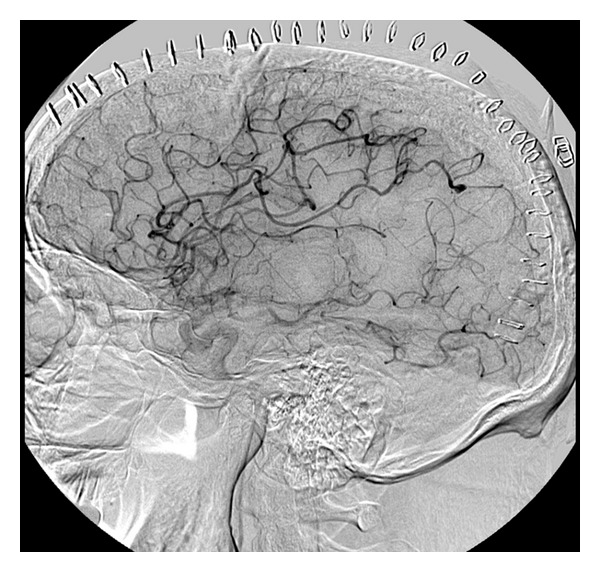
Carotid Cerebral Angiogram (Right Internal Carotid Artery injection, lateral view) revealing a 3 × 3 mm aneurysm on a distal angular MCA branch.

 Once infectious endocarditis was established, a diagnostic angiogram was performed to further investigate the etiology of the hematoma. Considerations included septic cardioemboli or a ruptured mycotic aneurysm. The angiogram revealed a solitary mycotic aneurysm in a distal segment of the right middle cerebral artery (Figure 4). He underwent successful glue embolization. The patient subsequently had a tracheostomy and percutaneous gastrostomy placed and was transferred to a skilled nursing facility. He continued to be in a vegetative state.

## 3. Discussion

IE may present with primarily neurologic symptoms. Yanagihara et al. presented a case of *Staphylococcus aureus* endocarditis presenting with fever and concurrent SDH and SAH [3]. In their patient, multiple abscesses were found on brain imaging; however, no mycotic aneurysm was isolated. Ruttmann et al. [4] reported a case of IE presenting with left-sided hemiplegia, and diagnostic evaluation revealed cardioembolic stroke. To date, we are unaware of any case report in which infectious endocarditis presented with intracranial hemorrhage and was diagnosed with positive evacuated hematoma cultures.

*Staphylococcus aureus* and *Streptococcus viridans* are the most common pathogens isolated from blood cultures in IE. *S. aureus, Enterococcus, and Escherichia coli* carry the worst outcome, related to their predisposition for multiple cerebral emboli. In contrast, emboli associated with streptococcal infections usually occur later, within the second week of infection. Streptococcal endocarditis is associated with a solitary embolus and carries a slightly more favorable prognosis [1]. Interestingly, although Streptococcus viridans is associated with rheumatic fever, our patient had no known history of this.

The relationship between mycotic aneurysms and intraparenchymal hemorrhage is well established. The most common location for mycotic aneurysms is the distal middle cerebral artery, while the second most commonly affected area is the cortical grey white junction [5]. The diagnostic imaging test of choice is a cerebral angiogram. However, the incidence of angiogram-negative hemorrhage with IE is high. In these circumstances, other causes must be considered, including hemorrhagic conversion and septic necrosis of the arterial wall. In patients with known infectious endocarditis presenting with new neurologic symptoms, an angiogram is indicated.

Prognosis in patients with neurologic complications associated with IE is based on early antibiotic therapy, as well as degree of neurologic injury. Initiating therapy earlier is associated with improved outcomes [6]. In our patient, antibiotic therapy was started at the prior hospital, although there was no complaint of fever. This may have contributed to the negative blood cultures seen on this admission. In addition, he did not have the usual sequelae of endocarditis, including Roth spots and Osler's nodes. IE located at the aortic leaflet may produce an “Austin Flint murmur,” heard during diastole. In this patient, no heart murmur was appreciated on cardiac auscultation. Investigation of bacterial endocarditis as a cause for the ICH was initiated by growth of Streptococcus viridans from the patient's evacuated hematoma.

## 4. Conclusion

We present a case of infectious endocarditis diagnosed from culture of the patient's evacuated intracranial hematoma.
